# SG-APSIC1120: Hand hygiene knowledge: Its effect on hand hygiene adherence rate during the COVID-19 pandemic in the primary care setting

**DOI:** 10.1017/ash.2023.51

**Published:** 2023-03-16

**Authors:** Chau Chain Yan, Giselle Li

**Affiliations:** National University Polyclinics, Singapore; Singapore National University Polyclinics, Singapore

## Abstract

**Objectives:** Good hand hygiene knowledge among healthcare workers (HCWs) is important in the fight against COVID-19. The coronavirus disease is primarily spread through droplet and contact routes, so hand hygiene and PPE are key infection control measures to protect patients and HCWs. We sought to determine whether hand hygiene knowledge scores had an impact on the hand hygiene adherence rate during a pandemic. **Methods:** Hand hygiene audit observations that were conducted covertly on a monthly basis and are presented as percentages of adherent reactions to moments to wash or sanitize hands. These data were examined in relation to HCW knowledge scores on hand hygiene. The knowledge scores on hand hygiene were analyzed based on 15 questions derived from WHO tools. Scores were determined using a quiz administered in a hand hygiene promotion event. **Results:** In total, 195 HCWs participated and scores on hand hygiene knowledge were ranked into 3 categories: 2% scored ≥90% (high), 60% scored 70%–89% (medium), and 38% scored ≤70% (low). Knowledge scores at the medium level and above were considered satisfactory. Even though 38% of the participants scored ≤70%, there was no direct impact on monthly hand hygiene audit observation rates in the 6 healthcare clinics. Hand hygiene observation rates ranged from 90% to 97%, with an overall mean of 92% for 2021. **Conclusions:** Contrary to studies that have shown the significant impact of knowledge on the hand hygiene adherence rate, our data suggest that a high hand-hygiene adherence rate is achievable and sustainable among HCWs. Adherence could be driven by attention to the importance of hand hygiene associated with the pandemic and potential exposure to COVID-19. High hand-hygiene compliance attains a place of importance in the minds of HCWs during a pandemic crisis.

